# Unexpected diversity among small-scale sample replicates of defined plant root compartments

**DOI:** 10.1038/s41396-021-01094-7

**Published:** 2021-11-10

**Authors:** Sally Attia, Jakob Russel, Martin S. Mortensen, Jonas S. Madsen, Søren J. Sørensen

**Affiliations:** 1 Section of Microbiology, Department of Biology, University of Copenhagen, Copenhagen, Denmark; 2 Department of Agricultural Microbiology, Faculty of Agriculture, Zagazig University, Zagazig, Egypt; 3 Host-Microbiota Interactions Laboratory, Wellcome Sanger Institute, Hinxton, UK

**Keywords:** Soil microbiology, Microbial ecology

## Abstract

Community assembly processes determine patterns of species distribution and abundance which are central to the ecology of microbiomes. When studying plant root microbiome assembly, it is typical to sample at the whole plant root system scale. However, sampling at these relatively large spatial scales may hinder the observability of intermediate processes. To study the relative importance of these processes, we employed millimetre-scale sampling of the cell elongation zone of individual roots. Both the rhizosphere and rhizoplane microbiomes were examined in fibrous and taproot model systems, represented by wheat and faba bean, respectively. Like others, we found that the plant root microbiome assembly is mainly driven by plant selection. However, based on variability between replicate millimetre-scale samples and comparisons with randomized null models, we infer that either priority effects during early root colonization or variable selection among replicate plant roots also determines root microbiome assembly.

## Introduction

It is broadly recognized that distinct assembly rules govern the establishment of microbiota around, on, and inside the plant root. There are three main compartments of the plant root that microbes occupy: rhizosphere, rhizoplane, and endosphere. These compartments harbor distinctive microbiomes for which the plant provides specific biotic and abiotic conditions [1]. Previous studies on plant root microbiomes have suggested a two-step selection process whereby microbiomes associated with root compartments become distinguished from the surrounding soil communities [2]. During this process, soil properties, vegetation history [3], and plant rhizodeposition have been found to be accountable for microbial enrichment in the rhizosphere soil [4, 5]. Then, during the second step, the host plant genotype influences and hereby fine-tunes the composition of the rhizoplane and endosphere communities [6, 7]. In addition, both the immune system [8, 9] and the developmental stage of the plant [10, 11] are key factors that influence the assembly of root-associated microbiomes.

Since the plant rhizosphere is a copiotrophic and dynamic environment, antagonistic and synergistic biotic interactions may both be significant drivers that shape community structure. These interactions can be strengthened by priority effects, whereby early colonizing microbes can determine which microbes are able to colonize later [12, 13]. To better understand whether interactions play a decisive role in the assembly of plant root microbial communities, they need to be investigated at a resolution, whereby the scale of sampling is both small enough to be relevant for identifying microbial interactions and stochastic processes, yet, large enough to capture ecosystem processes [14, 15]. By implementing millimetre-scale sampling, at which spatial patterning occurs for individual root-associated communities, it should be possible to get insight into the complexity of the underlying mechanisms accountable for structuring root-associated bacterial communities. However, despite the use of small-scale samples, one cannot rule out that replicate plant roots could select for different microbes, thus obfuscating the inference of interactions through co-occurrence.

There is rich literature examining the root microbiome composition at the scale of the whole root system, for example [16–18]. However, understanding to what extent local dynamics of different types of root systems can explain the patterns of community assembly remains elusive. Here, we adopted a fractionation protocol to separate the rhizosphere and rhizoplane bacterial communities in replicated small-scale samples. We extracted bacteria from millimetre-scale subsections of the cell elongation region of individual roots (5 mm), both from wheat and faba beans crops, to investigate the relative importance of deterministic and stochastic processes on root microbiome assembly. Specifically, we assessed (i) the role of environmental factors (plant species and soil type) in shaping beta-diversity patterns in the soil- and root-associated bacterial community and (ii) the potential influence of microbial interactions.

## Material and methods

### Experimental set-up

Two plant growth experiments were performed under greenhouse conditions to profile the rhizosphere and rhizoplane-associated bacterial community structure of wheat and faba bean crops, and to investigate mechanisms affecting microbial community assembly. Soil samples were gathered from two agricultural fields located in Taastrup, Denmark. Field 1 has a long-term history; 7 years of cultivation wheat crops, whereas field 2 has a shorter history; 2 years of growing faba bean crops. Both soil types were transported to a greenhouse, separately air-dried, passed through a 2 mm sieve, and filled into square pots (10 × 10 cm; 400 g soil/pot), each in 15 replicates. Ten sterilized and sprouted wheat seeds were seeded in each wheat soil-filled pot. Five faba bean seeds were seeded in each of the pots with faba bean soil. All pots received 10 ml of Hoagland solution (NPK fertilizer), once before planting the seeds, and again 15 days after. After 1 week of growth, the germinated seeds were thinned to three plants per pot. Three soil samples of each field were used as soil biome controls. Control samples were collected, air-dried under the greenhouse conditions for 3 days, and then subjected to DNA extraction. The experiments were carried out under controlled greenhouse conditions with photoperiods of 16 h/21 °C days, 8 h/16 °C nights, and regularly irrigated with tap water. Both plant species were harvested 21 days after sowing: wheat plants were at tillering stage (three leaf, with established primary, fibrous root system) and faba bean plants were at growth stage (three leaf, 5 node stage, with established taproot system).

### Sampling of rhizosphere and rhizoplane-associated microbial communities

For both plant species, one healthy plant from each pot was uprooted entirely with its surrounding soil at the seedling stage. Excess soil was manually separated from the roots by shaking, leaving an ~1 mm thick layer of soil still attached to the roots. Next, 5 mm of the cell elongation zone was sampled from a single root of each individual plant. In total 15 root segments were collected for each plant species. To extract the rhizosphere and rhizoplane bacteria from the same root segment, we used a fractionation and detachment protocol [19]. Briefly, to collect the rhizosphere suspensions (Rs), root segments were separately placed into micro-centrifuge tubes containing 1 mL of PBS and shaken at 300 rpm for 15 min at 4 °C. Next, the same root segments were washed two times in fresh PBS, transferred to new micro-centrifuge tubes containing 1 mL PBS, and then subjected to sonication using a sonication bath for 1 min at 4 °C, to collect the rhizoplane suspensions (Rp).

### DNA extraction and PCR amplification

Genomic DNA was extracted from 250 mg of each soil sample and 150 μL of each rhizosphere and rhizoplane suspensions using a FastPrep-24 bead-beating system (MP Biomedicals at 5 m/s for the 30 s) and the NucleoSpin Soil Kit (Macherey-Nagel, Germany) following the manufacturer’s instructions. The DNA of all samples was eluted in 30 μL elution buffer (5 mM Tris/HCl, pH 8.5), and the extracted DNA was stored at −20 °C.

The 16S rRNA gene amplification procedure was divided into two PCR steps. In the first PCR reaction, the extracted DNA was amplified by using the modified broad range primers Uni341F (5″-CCTAYGGGRBGCASCAG-3′) [20] and Uni806R (5″-GGACTACHVGGGTWTCTAAT-3′) [21] that amplifies the hypervariable V3-V4 regions of 16S rRNA genes (~466 bp). PCR reactions were performed in 96-well microtiter plates, using PCRBIO HiFi polymerase (PB10.41, PCRBIOSYSTEMS, UK) modified to 25 μL reactions (2 µL DNA template, 5 µL reaction buffer, 1 µL of each forward and reverse primer, 0.25 μL polymerase, 15.75 μL molecular grade water [W4502, Sigma, UK]), following manufacturer’s instructions. Reactions were run in a 2720 thermal cycler (Applied Biosystems, Life Technologies, CA, US). For each plate, a negative template-free control and a positive control containing 2.0 μL DNA from a known bacterial mock community (1.0 ng/μL; HM-782D, BEI Resources, VA, US) were included. Agarose gel electrophoresis was used to verify successful amplification. In the second step, sequencing primers and adaptors were added to the amplicon products. Primers developed in-house were used that contains sequencing adaptors and unique combinations of forward and reverse indices [22]. The amplicon PCR products were purified using Agencourt AMPure XP (Beckman Coulter, USA) PCR Clean-Up System (13 μL AmPure beads: 20 μL PCR product) as recommended by the manufacturer.

### 16S rRNA gene amplicon sequencing

Samples were normalized using the SequalPrep Normalization Plate (96) Kit (Invitrogen, Maryland, MD, USA), concentrated using the DNA Clean and Concentrator-5 kit (Zymo Research, Irvine, CA, USA). The concentration of the pooled libraries was determined using the Quant-iT High-Sensitivity DNA Assay Kit (Life Technologies) following the specifications of the manufacturer and adjusted to 1.65 ng/μL (4 nM). Amplicon sequencing was performed the MiSeq System (Illumina Inc., CA, US), with the denatured libraries adjusted to a final concentration of 16 pM. For each run, a 5.0% PhiX internal control was included. All reagents used were from the MiSeq Reagent Kits v2 (Illumina Inc., CA, US). Automated cluster generation and 250 paired-end sequencing with dual-index reads were performed. The sequencing output was generated as a demultiplexed fast Q-file for downstream analysis. Up to 192 samples, including controls, were sequenced per run.

### Bioinformatics analysis

Primers were removed from the raw paired-end FASTQ files generated via MiSeq using Cutadapt [23]. Raw sequence data were processed by QIIME2 [24] pipeline using DADA2 [25] to infer amplicon sequence variants (ASVs) present and their relative abundances across the samples. Forward and reverse reads were trimmed at the 5′ end until 8 bp; other quality parameters used DADA2 default values. Taxonomy was assigned using a pre-trained Naïve Bayes classifier (Silva Ref NR 99 [release 132] [26]).

### Statistical analysis

All analyses were run on a non-rarefied dataset (Fig. S1, [27]). We have also run the analysis on a rarefied dataset with 1801 reads/sample to assure that sampling depth did not impact our conclusions. Both methods yielded qualitatively identical results (Fig. S2, Fig. S3, and Fig. S4). We, therefore, present the analysis of the non-rarefied data as it preserves more information. Also, in our primary analyses, beta Raup-Crick and the correlation networks, we compare against null models with similar richness to the observed data, thereby minimizing sampling-depth bias.

We performed all data analyses in R version 3.3.6 (R Core Team 2019). To assess bacterial variation within each sample (alpha diversity) we used two estimates: (1) Observed richness and (2) Shannon index using the package ‘vegan’ version 2.5–4 [28]. Wilcoxon rank-sum test was used to test the difference in richness between rhizosphere, rhizoplane, and soil samples. To investigate the patterns of beta diversity we used phylogenetic-based metrics; weighted and unweighted UniFrac distances and the count-based metric Bray–Curtis dissimilarity. Principal coordinate analysis (PCoA) was used to visualize the dissimilarity matrices. We tested for significant variation between the bacterial communities’ structures of the sample types by Permutational analysis of variance (PERMANOVA [29]) for the various beta diversity metrics, using the vegan function “adonis” with 999 permutations. To identify the bacterial taxa accountable for the divergence among the root compartments and the soil samples, we used the DAtest package (version 2.7.11 [30]) with default options. The best performing methods for faba bean and wheat were EdgeR quasi log-likelihood [31] with TMM (erq) and RLE (erq2) normalizations, respectively. To investigate whether the composition of the microbiome might be assembled by stochastic or by deterministic factors, we applied a modified Raup-Crick dissimilarity metric “βRC” as implemented in the function “raupcrick” from the vegan package [32]. The function “raupcrick” treats the data as binary (presence/absence data). The null hypothesis is based on null communities created by random sampling of taxa with the sample richness similar to the observed sample richness. The null models were constrained within each group of samples. The results are a probability for each pair of samples of whether they are non-identical. To explore co-occurrence between bacteria, we calculated proportionality between ASVs to infer potential pairwise associations between them [33]. Fifteen samples of each root compartment of both plant species were included in the co-occurrence analysis. Only ASVs with more than 50 reads in total were included. Correlations were considered if the absolute value of the correlation coefficient (*r*) was >0.6. Similar to the Raup-Crick analysis we created null models (99) constrained by the sample richness using “permatfull” [28] and calculated the proportionality for each similar to the observed networks. The number of edges in the observed network was then compared to the number of edges found in the null model networks. Networks were graphed and visualized using Cytoscape 3.7.2 [34].

## Results

### Plant compartments differ from each other and from the soil

Proteobacteria and Bacteroidetes were enhanced in the wheat rhizosphere and rhizoplane compared to wheat soil samples. Whereas, both faba bean compartments consisted mainly of Proteobacteria, Bacteroidetes and Verrucomicrobia, and to a lesser extent, Patescibacteria (Fig. S5). Contrariwise, Acidobacteria and Firmicutes dropped in the faba bean compartments compared to faba bean soil, and Acidobacteria, Actinobacteria, and Thaumarchaeota in the wheat compartments compared to wheat soil.

**Fig. 1:**
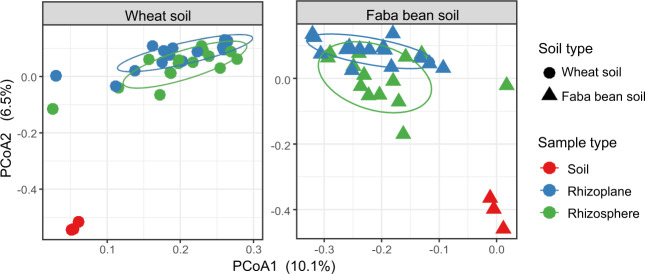
Replicate small-scale samples show variation in the root-associated microbiome and much divergence from the soil. The experimental variables were colored by sample type (Soil, Rhizosphere and Rhizoplane of wheat and faba bean plants) and shaped by soil types (wheat and faba bean soils); the ellipses indicate 75% confidence regions for clusters.

To investigate the distinction between microbiomes of the different plant rhizocompartments and the soil types, we performed PCoA based on Bray–Curtis dissimilarity. The PCoA revealed a clear separation between the soil and the root-associated microbiomes, with an apparent clustering of bacterial communities according to their host root compartments (Fig. 1). PERMANOVA results indicated that the plant types had the largest impact on bacterial community composition compared to whether it was a soil or root sample (*R*^*2*^ = 0.087; *p* < 0.001 and *R*^*2*^ = 0.058, *p* < 0.001, respectively, Table S1). Noticeably, clustering of the faba bean rhizosphere and rhizoplane samples was significantly apparent, but not for the wheat samples. Likewise, a PCoA based on weighted and unweighted UniFrac distances (Fig.S6) confirmed the observed differentiation between the root-associated assemblages and the soil communities. In support, the results of PERMANOVA using both metrics confirmed the variation between soil and root communities (*p* < 0.001; Table S1).

To further explore which bacterial taxa were accountable for the divergence among the root compartments and the soil samples, we used differential abundance analyses (see Methods and Materials for details). These showed that *Massilia, Acidovorax, Pseudomonas, Fluviicola*, and *Cutibacterium*, constituted a conserved wheat microbiome whose enrichment differentiated the wheat root compartments from the wheat soil [EdgeR qll - RLE (erq2), *p*.adj < 0.05; Fig. S7A]. Only, *Phaselicystis* were enriched in the rhizosphere and *Stenotrophomonas* in the rhizoplane. In contrast, the enrichment of *Staphylococcus* and *Enterococcus* in the faba bean rhizoplane and *Ca-Xiphinematobacter* in the rhizosphere significantly discriminated between root compartments and faba bean soil [EdgeR qll - TMM (erq), *p*.adj < 0.05; Fig. S7B]. Also, *Methylophilaceae* and *Geothrix* were enhanced in both root compartments compared to the faba bean soil.

**Fig. 2:**
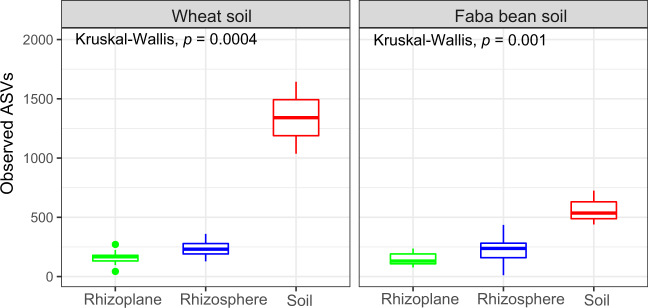
A significant reduction in the level of bacterial richness in rhizosphere and rhizoplane of wheat and faba bean compared to that of the soil samples. Boxplot of observed richness by plant and sample type. Significance determined by the Kruskal–Wallis rank-sum test and Wilcoxon–Mann–Whitney pairwise comparison test.

Inspection of the alpha diversity of the wheat and faba bean root-associated microbiomes illustrated a significantly reduced bacterial richness (Fig. 2) and diversity (Fig. S8) in the root microbiomes compared to that of the soil (Wilcoxon Rank-Sum test, *p*.adj < 0.001).

Collectively, our findings confirm that the root compartments are colonized by a taxonomically distinct bacterial community, showing the importance of the plant root microenvironment on the selection of the plant root microbiome [2].

### Heterogeneity between small-scale samples of rhizocompartments is non-random

Interestingly, there was a noticeably large variation among the individual rhizocompartments replicate samples, while replicate soil samples were much more uniform (Fig. 1). We, therefore, tested to what degree this variance between small-scale samples was a result of deterministic factors such as microbial interactions or simply could be explained by stochasticity, such as technical variation during sampling.

**Fig. 3:**
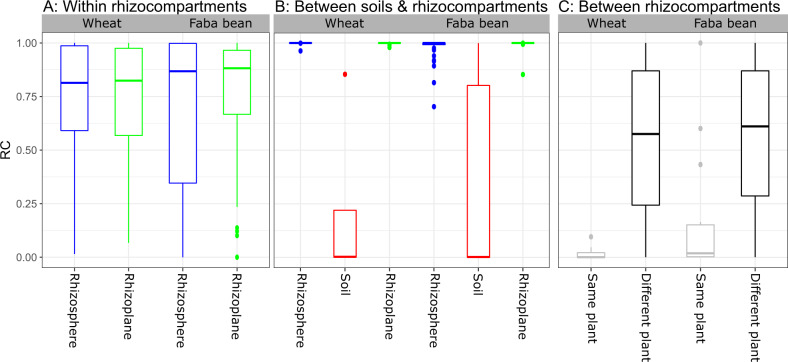
Heterogeneity between small-scale samples of rhizocompartments is non-random. βRC closer to zero indicates that stochastic processes are more important, whereas βRC closer to 1 indicates that deterministic processes (e.g., environmental selection and bacterial interactions) are more important. **A** Shows that variability among individual replicate samples within the rhizosphere and the rhizoplane of both plant species is non-random. **B** Shows deterministic environmental selection by the plant on the root microbiome. **C** Shows the effect of stochastic processes on bacterial assembly between rhizosphere (Rs) & rhizoplane (Rp) from the same plant and different replicate plants of the same plant type, which belong to the same plant type. The null models were constrained within each group of samples (15 replicates/group).

To better understand the cause of the observed variance, we investigated the patterns of the microbiome assembly using a modified Raup-Crick dissimilarity metric [32], referred to as βRC [35]. This metric defines the relative magnitude of the dissimilarity between real observed communities and those expected by chance produced by a null model approach [36]. For this, we used null models with similar richness as the observed communities, and the null models were constrained within each group of the samples (Fig. 3).

For both plant species, this approach revealed that the βRC values of assembled communities within each of the rhizosphere and rhizoplane samples (βRC~0.9) deviated from the null expectation. This indicates that the observed communities in replicate samples were more different from each other than expected by chance (Fig. 3A). When comparing the soil to the root compartments (Fig. 3B) the βRC values (βRC = 1) were also significantly different from the null expectation, suggesting that the distinct root microbiota were more different from the soil community than expected by chance. In contrast, βRC did not differ from the null model between the rhizosphere and rhizoplane compartments for the same plant, indicating a role of stochastic processes in structuring communities between the rhizosphere and rhizoplane compartments originated from the same piece of root from the same plant (Fig. 3C).

### Bacterial co-occurrences are enhanced in the rhizosphere and rhizoplane

Lastly, we analyzed bacterial co-occurrence and co-exclusion patterns within each root compartment, to further investigate the observed non-random difference in community structure between replicate small-scale samples.

**Fig. 4:**
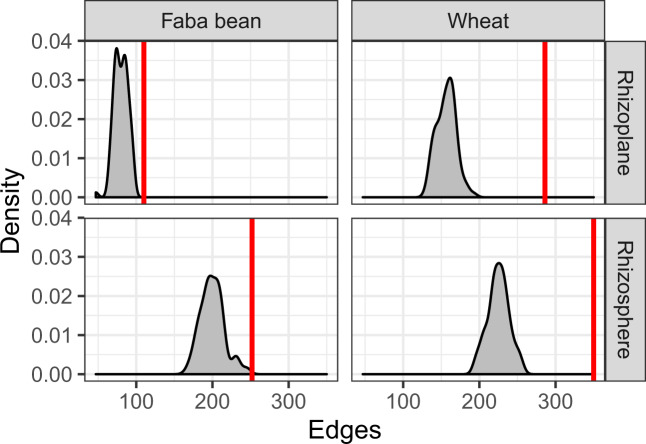
Non-random co-occurrences are prevalent within bacterial communities associated with the rhizosphere and rhizoplane of wheat and faba bean crops. The *x*-axis shows the number of edges (connections in networks). The red line is the number of edges in the observed networks. The gray density plot is the distribution of edges from null models. There are more edges in the real observed networks than that of the null models, indicating that the patterns of co-occurrence and co-exclusion within each network are not random.

We found that patterns of bacterial co-occurrence within each root compartment varied in their complexity and organization, in which network complexity and connectivity decreased with the proximity to the host plant. Hence, the rhizosphere networks showed more complexity and connectivity as they had more nodes and edges, and fewer connected components than those of the rhizoplane networks of both plant species (Supplementary Table S2: Topology network analyses). We tested whether co-occurrence happened stochastically or non-randomly by comparing the number of edges in the observed networks to those expected in random communities (null model). We found that there were more edges in the observed network than in any of the null models, particularly in the wheat rhizosphere and rhizoplane networks, indicating that the patterns of co-occurrence and co-exclusion within each root compartment were non-random (Fig. 4, Fig. S9, and Fig. S10).

## Discussion

The focus of this study was to understand what processes influence local microbiome assembly on different types of root systems, particularly, the importance of bacterial interactions on the root-associated microbiome assembly.

### Strong selective effects by host plant species on the root-associated microbiome composition

Our results revealed that the plant rhizocompartments house distinct microbiomes with taxonomic profiles that differ from those in soil. In accordance with recent studies reporting an influence of the plant root compartments on the microbiome composition, for example [37, 38]. We found a significant reduction in bacterial richness and diversity from the soil to the rhizocompartments. This confirms that host plants exert selective effects on the soil microbiota [39, 40] and we found these effects to be the most important driver of the assembly of root-associated communities in both fibrous and taproot model systems, represented by wheat and faba bean. The host plants can influence the root habitat-types in different ways [41]. For example, plants interact with microbes through the rhizodeposition process, which attracts and boosts a subset of the soil microbiota, that has the metabolic capacity to exploit the root exudates [40, 42–44]. It has thus been suggested that root exudates, in addition, mediate the interplay between roots and microbes and among microbes at the initial events of colonization [45–47]. In addition, variation in root exudation components can result in differences in composition and microbial abundances of root-associated microbiota [48].

### High variability among small-scale replicate samples

Interestingly, the observed microbial communities among individual replicate samples within each root compartment were more different from each other than expected by random chance (βRC~0.9; Fig. 3A). The individual plants were grown under homogeneous environmental conditions (plants were grown in their co-adapted soil under uniformly controlled conditions), which implies that the selective environmental effects on the soil microbial communities will also be homogeneous [49]. Hence, our results suggest that heterogeneous selective processes create variation between replicates. One mechanism could be that even if environmental conditions are homogeneous through space, the random order and timing of species arrival into a given locality, which is known as priority effects [13], can lead to very dissimilar community composition even when all species have access to the community [12]. It has been argued that the large diversity in soil microbial communities may present plants with sufficient variation in the species pool of root microbiota to create individual patterns, even at a local scale [42]. However, our data did not support this notion, since this, all else being equal, would result in random assemblies. We observed non-random co-occurrence patterns within each root compartment, indicating that rhizosphere and rhizoplane bacterial subcommunities tend to co-occur more than expected by chance. Although co-occurrence and co-exclusion among microbes is not a measure of their interactions, the non-random variation among replicates could suggest a role of microbial interactions in the root microbiome assembly. We note that co-occurrence can have many underlying causes, interactions being only one of these [50]. Alternatively, there could be variations among the replicate plants, which for example, through the plant rhizodeposition processes could drive the observed variations among replicate samples. Considering all the evidence presented here jointly, deterministic factors such as microbial interactions and environmental selection appear to be important drivers for the assembly of local small-scale root microbiomes.

### Stochasticity contributes to variability among specific rhizocompartments associated microbiota

The variation between the rhizosphere and rhizoplane bacterial communities of faba bean (*P* = 0.001, PERMANOVA) did not differ from the null expectation (βRC~zero, Fig. 3C), which indicates that faba bean rhizoplanes are a random subset of the rhizosphere communities. Bacterial communities associated with the wheat rhizocompartments were not significantly different and were also not differing from the null expectation (βRC~zero, Fig. 3C). Similarly, this suggests that for wheat the rhizoplane communities are a random subset of the rhizosphere communities.

## Conclusion

Overall, we found that environmental selection by plant species strongly determines local community composition of plant rhizocompartments, and that roots at a small scale show surprising microbiome variation, which likely was driven by bacterial interactions or environmental variation among replicate plant roots.

## Supplementary information

Supplementary Material

## Data Availability

The data for this study have been deposited in Sequence Read Archive (SRA) under the Bioproject ID: PRJNA744195 (https://www.ncbi.nlm.nih.gov/bioproject/744195).
