# Supplementary material for: Unexpected diversity among small-scale sample replicates of defined plant root compartments
Source: ISME J. 2021 Nov 10;16(4):997–1003. doi: 10.1038/s41396-021-01094-7 (PMC8940884; doi:10.1038/s41396-021-01094-7)
Supplement: Supplementary file 1 — Supplementary Material [file 41396_2021_1094_MOESM1_ESM.docx]

**Supplementary Figures**


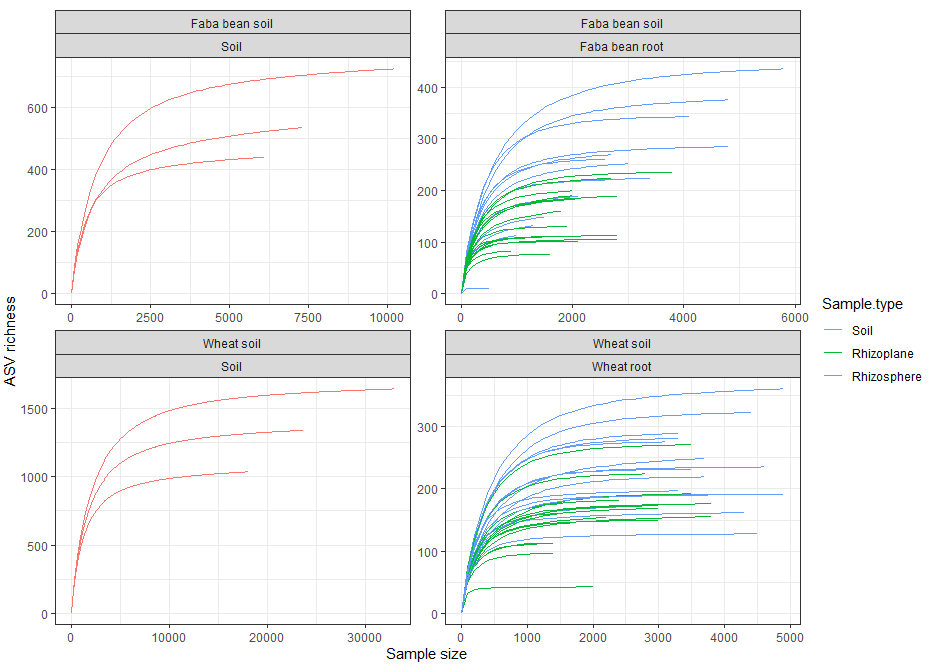


**Figure S1:** Rarefaction curves for all samples. Observed ASV richness plotted against a range a subsampled sequencing depth. Each line is a sample.

**Figure S2:** Observed ASV richness on rarefied data.

**Figure S3:** Principal coordinate analysis (PCoA) based on Bray-Curtis dissimilarity on rarefied data.


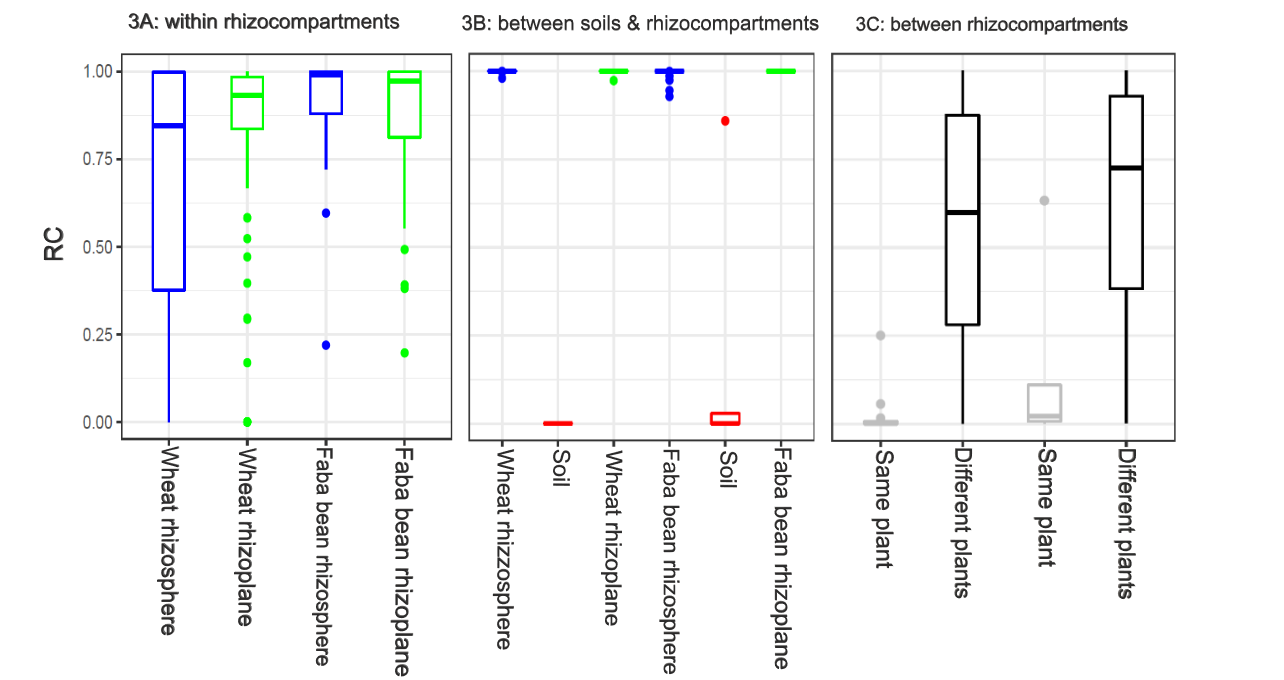


**Figure S4:** βRC analysis on rarefied data


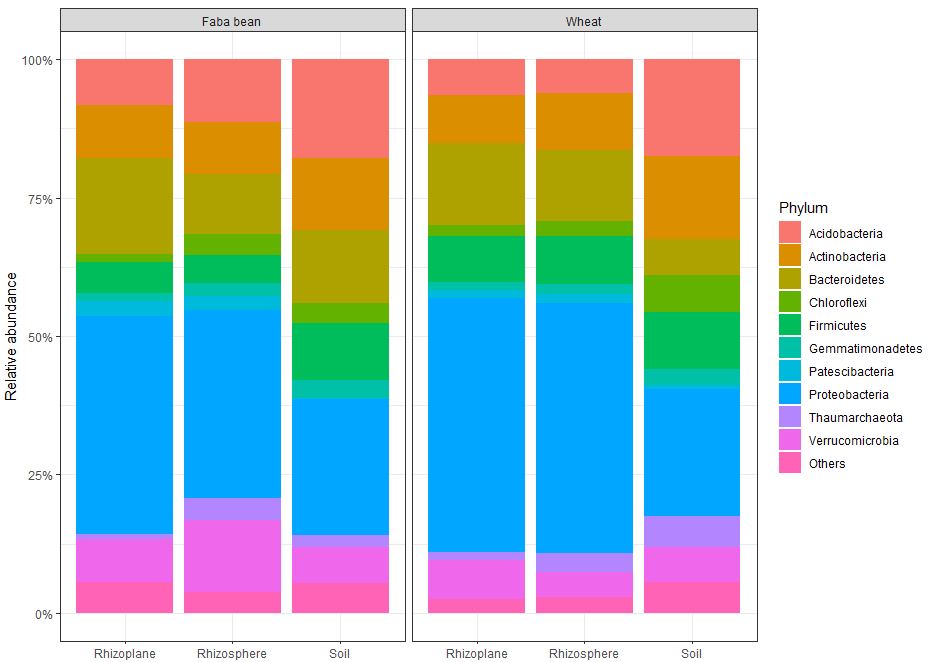


**Figure S5:** Bar plot depicting the relative abundance of the microbial phyla associated with the wheat and faba bean rhizosphere, rhizoplane, and soil samples. The 10 most abundant phyla are colored separately, and the remainder are grouped in the “Others” category.

**Figure S6:** Phylogenetic dispersion between the root-associated microbiome and that of the soil. Principal Coordinate Analysis (PCoA) of the wheat and faba bean roots microbiome based on weighted (S6A) and unweighted (S6B) UniFrac distances analyzed to investigate the overall change in bacterial community among all experimental groups. The testing groups were shaped by sample type (Soil, Rhizosphere and Rhizoplane of both plant species), and colored by soil types (wheat and faba bean soils). The ellipses indicate 75% confidence regions for group clusters.


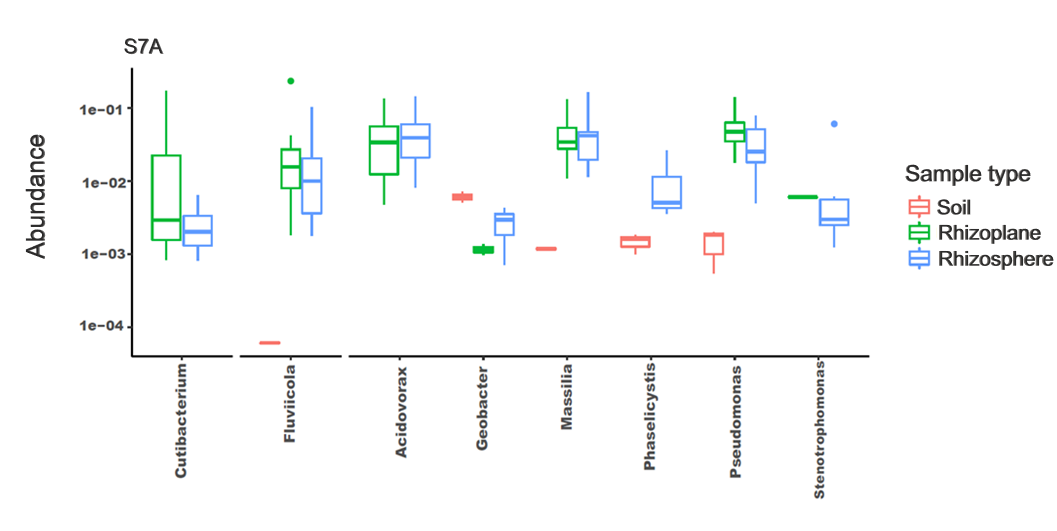


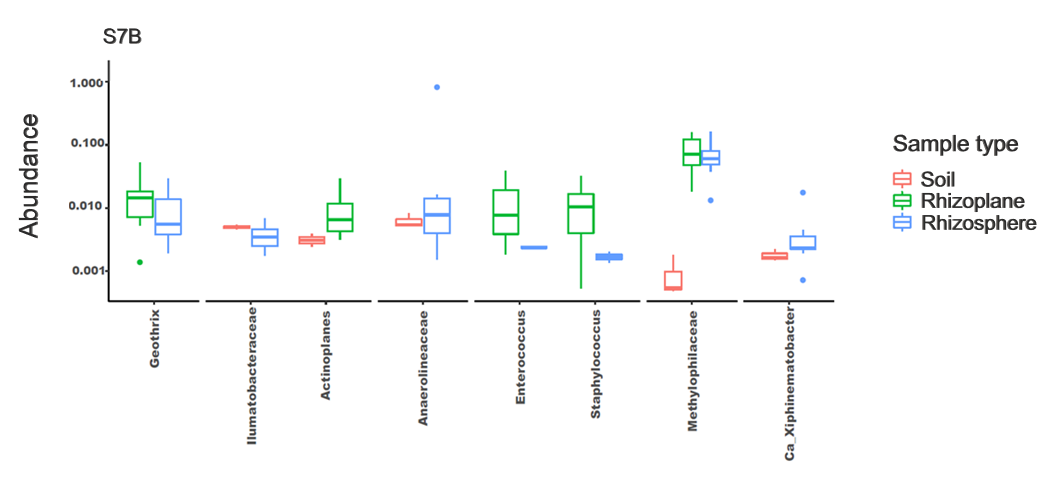


**Figure S7:** Boxplot of relative abundance of species significantly associated with rhizosphere, rhizoplane, and soil samples of Wheat (S7A) and faba bean (S7B). Y axis is plotted on log scale.

**Figure S8:** Boxplot of alpha diversity estimated by Shannon index shows the level of bacterial diversity in the wheat and faba bean rhizosphere and rhizoplane. Significance determined by the Kruskal-Wallis rank-sum test and Wilcoxon–Mann–Whitney pairwise comparison test.

**Figure S9**: Network of co-occurred and co-excluded ASVs within the rhizosphere (S9A) and rhizoplane (S9B) of faba beans. A correlation analysis stands for a strong (|r| > 0.6 and significant P < 0.01) correlation. The size of each node is equivalent to the number of connections (Node degree). Edges connecting two nodes represent the interactions between them. Blue lines represent the positive significant correlations, and red lines represent a negative significant correlation. The identity of ASVs was omitted for simplicity.

**Figure S10**: Network of co-occurred and co-excluded ASVs within the rhizosphere (S10A) and rhizoplane (S10B) of wheat. A correlation analysis stands for a strong (|r| > 0.6 and significant P < 0.01) correlation. The size of each node is equivalent to the number of connections (Node degree). Edges connecting two nodes represent the interactions between them. Blue lines represent the positive significant correlations, and red lines represent a negative significant correlation. The identity of ASVs was omitted for simplicity.

**Supplementary Tables**

**Table S1:** Result of PERMANOVA analyses with 999 permutations based on Bray-Curtis, weighted and unweighted UniFrac dissimilarity matrix.

|  | Bray-Curtis | | Weighted UniFrac | | Unweighted UniFrac | |
| --- | --- | --- | --- | --- | --- | --- |
|  | *R^2^* | Pr(>F) | *R^2^* | Pr(>F) | *R^2^* | Pr(>F) |
| Root/Soil (WR & FR vs Soils) | 6% | 0.001*** | 8% | 0.001*** | 5% | 0.001*** |
| Soil type (Ws & Fs) | 9% | 0.001*** | 12% | 0.001*** | 4% | 0.001*** |
| Root/Soil*Soil type | 3% | 0.002** | 2% | 0.1 | 2% | 0.001*** |

**Table S2:** Topological Properties of the rhizosphere and rhizoplane networks of both plant species

| **Network properties** | **Faba bean** | | **Wheat** | |
| --- | --- | --- | --- | --- |
|  | **rhizosphere** | **rhizoplane** | **rhizosphere** | **rhizoplane** |
| Nodes | 110 | 79 | 158 | 123 |
| Edges | 246 | 103 | 357 | 286 |
| Positive interactions | 155 | 78 | 311 | 253 |
| Negative interactions | 91 | 25 | 46 | 33 |
| Connected components | 2 | 7 | 2 | 5 |
| Density | 0.041 | 0.033 | 0.029 | 0.038 |
| Heterogeneity | 0.621 | 0.512 | 0.617 | 0.798 |
| Diameter | 11 | 13 | 12 | 12 |
| Centralization | 0.061 | 0.045 | 0.061 | 0.086 |
| Clustering coefficient | 0.287 | 0.337 | 0.353 | 0.327 |
| Characteristic path length | 4.050 | 4.894 | 4.809 | 4.907 |
| Neighborhood connectivity | 4.473 | 2.608 | 4.519 | 4.650 |
